# Supplementary material for: Aerobic capacity and muscular strength in 179 Norwegian men and women aged 20–59 years with a substance use disorder: A cross-sectional study
Source: PLoS One. 2026 May 13;21(5):e0348707. doi: 10.1371/journal.pone.0348707 (PMC13170891; doi:10.1371/journal.pone.0348707)
Supplement: Attachment — Submitted filename: review report .docx [file pone.0348707.s003.docx]

**Manuscript ID:** PONE-D-25-47064R1
**Title:** *Aerobic capacity and muscular strength in 179 Norwegian men and women aged 20–59 years with a substance use disorder: A cross-sectional study*

The revised manuscript substantially addresses the concerns from the first review round and is now suitable for PLOS ONE with ***minor*** revisions.

**Strengths**

The authors have made meaningful improvements across all major points raised previously. They now report that 207 patients were eligible, 28 declined participation (87% inclusion rate), and all 179 completers finished testing without adverse events—this transparency helps readers better assess selection bias and safety. The new HUNT3 reference section (with Table 2) clearly outlines protocol similarities/differences, making the fitness comparisons more interpretable. Confidence intervals appear in Tables 4-5, effect sizes (η²) are reported for age-group ANOVAs, and scatterplots visualize variability effectively. The added regression (Table 6) adjusting for age, BMI, and smoking strengthens the analysis, though its limitations due to unavailable medical-record data (e.g., medications, comorbidities) are appropriately acknowledged. Conclusions remain appropriately descriptive for the cross-sectional design.

**Remaining issues**

**Data availability**: The commitment to share raw data "freely if accepted" aligns with PLOS ONE policy in principle, but lacks specifics on repository (e.g., Figshare, Zenodo), DOI, or timeline—please clarify this before acceptance to fully comply.

**Minor edits**:

- Table 4 (and likely Table 5 for women) needs reformatting for clarity—ensure columns align properly, full data visible on one page if possible, and consistent CI notation (e.g., 45.9 [44.5–47.3])
- Some phrasing remains awkward (e.g., "Patients that fulfilled the inclusion criteria, and agreed to participate, was tested" should be "were tested"; "oxygen pumpedheartbeat" needs spacing/formatting).
- Ensure consistent formatting for CIs and effect sizes across text/tables (e.g., η² values like "20.329 0,327" appear malformed).
- The CPET ventilation note (two participants >180 L/min) is clear but could be more concise.

**Recommendation**

**Minor revision**. These are straightforward fixes that do not affect scientific validity. The study provides valuable reference data on aerobic capacity and strength in SUD patients, filling an important gap. Once addressed, the paper will be ready for publication.
