# Supplementary material for: Aerobic capacity and muscular strength in 179 Norwegian men and women aged 20–59 years with a substance use disorder: A cross-sectional study
Source: PLoS One. 2026 May 13;21(5):e0348707. doi: 10.1371/journal.pone.0348707 (PMC13170891; doi:10.1371/journal.pone.0348707)
Supplement: Attachment — Submitted filename: Response to Reviewers.docx [file pone.0348707.s004.docx]

**Dear Editor,**

Thank you for your patience with our delay, and for giving us the opportunity to revise and re-submit an improved version of our manuscript. All comments and concerns from the editor and reviewers have been addressed with corresponding amendments in the manuscript. We thank you and the reviewers for valuable comments, surely making the manuscript better. We do hope that the revised version of the manuscript is satisfactory for publication in PLOS ONE.

Sincerely,

Henrik Loe

**Response to Journal and Reviewers:**

Journal:

The amended role of funder statement is now included in the cover letter as requested.

Thank you for the information on the potentially time consuming data availability process. We prefer to make the data freely accessible if the manuscript is accepted for publication.

**Reviewer #1:**

Thank you for taking the time to review our manuscript. We believe that your suggestions and comments have contributed to improve it. Please find the changes highlighted in the revised version of the manuscript.

- Addressing your concern regarding data availability: In line with PLOS ONE guidelines, all individual raw data underlying the results will be made freely available if the manuscript is accepted for publication. Data Availability Policy, so that all statistical analysis can be scrutinized.
- Response to your input on clinical implications. This is clearly a relevant and important factor and has now been addressed in an added section under the Discussion (lines 554-563)

**Reviewer #2:**

Thank you for your comprehensive feedback. Your comments and concerns are hopefully addressed adequately. Please find the changes highlighted in the revised version of the manuscript.

**2. Major Comments**

1. Selection bias and representativeness

**Comment:** Because participation required maximal testing, the sample may represent the fitter segment of the SUD inpatient population. Please provide the number of screened, excluded, or non-participating individuals and, if possible, basic characteristics (e.g., age, sex, BMI) to help readers assess representativeness.

**Reply:** Those who declined to partake when eligible for participation (Non-participants) and inclusion rate are now included under the heading: Settings and participants in the methods section (Lines 88-93). Unfortunately, basic characteristics were not collected from those who declined.

As you point out, participants may represent the fitter segment. This has been noted in the strength and limitation section (Lines 573-575), and in the discussion with a brief elaboration of consequences (Lines 543-550).

2. Statistical adjustment for confounders

**Comment:** The analyses are largely descriptive. Consider including regression models that adjust for potential confounders (age, BMI, smoking, type/duration of substance use, medications, comorbidities). Even simple adjusted models would enhance analytical rigor.

**Reply:** Thank you for this notice. We agree that a regression analysis for the key variable VO_2max_ will improve analytical rigor. This has been included in a new table (Table 6) in the Results section (Lines 306-311). This analysis is also described in the Statistical analysis section (Lines 218-219). Unfortunately, we did not have access to the patients’ medical records and were therefore unable to assess individual substance use, medication, or comorbidities.

3. Limited subgroup power

**Comment:** Some subgroups (notably older women) are small. Report confidence intervals and effect sizes to aid interpretation and explicitly discuss this limitation.

**Reply:** Confidence intervals are now reported in the revised version of table 4 and 5. Overall effect sizes from the one-way ANOVA are reported at the end of the paragraphs describing “age group differences” in the results section (Lines 268-269 and 291-293).

4. Comparability of reference values

**Comment:** The use of the HUNT3 dataset as reference data is appropriate geographically, but readers need clarity on differences in testing protocols, calibration, and population characteristics. A brief summary table or paragraph would help contextualize the comparisons.

**Reply:** Thank you for this suggestion. This has been rectified in a new paragraph in the Methods section named HUNT 3 reference material (Lines 147-154). We have also provided a new table (Table 2) summarizing basic characteristics (age group, sample size, VO2max, body mass, R-value).

5. Data availability statement

**Comment:** PLOS ONE requires data to be available at publication unless legal or ethical barriers apply. Please clarify exactly how and when data will be shared (e.g., through an anonymized repository, with DOI or controlled access).

**Reply:** In response to data availability. If the manuscript is accepted for publication all individual raw data underlying the results will be made freely available to be in compliance with the PLOS ONE Data Availability Policy, so that all statistical analysis can be scrutinized.

6. Adverse events and test completion.

**Comment:** Given the maximal CPET and 1RM protocols, please report whether any participants failed to complete testing or experienced adverse events, and how these cases were handled.

**Reply:** No adverse events occurred and everyone who partook completed tests. This is now specified under the heading: Settings and participants (Lines 88-93) in the Methods.

7. Instrumentation note.

**Comment:** The manuscript mentions a 5.5% error at very high ventilation rates (VE > 180 L/min). Indicate how many participants this affected and whether this could meaningfully influence results.

**Reply:** Two participants exceeded the predefined acceptance for error in the highest minute ventilations. This is now addressed in the Methods in the CPET section (Lines 138-140).

**3. Minor Editorial Comments.**

**Comment:** Report 95% confidence intervals and effect sizes (Cohen’s d, η², etc.) for key comparisons.

**Reply:** Amended as suggested. As noted in our previous reply, confidence intervals are now reported in the revised version of table 4 and 5. Overall effect sizes from the one-way ANOVA are reported at the end of the paragraphs describing “age group differences” in the results section (Lines 268-269 and 291-293).

**Comment:** Verify assumptions of normality and variance homogeneity; note if any nonparametric approaches were used when appropriate.

**Reply:** The assumption of normally distributed data was supported by QQ-plots, and variance homogeneity by Levene’s test. No nonparametric approaches were applied. This information is now added in the Statistical analysis section (Lines 211-212).

**Comment:** Streamline sections of the discussion to reduce redundancy and focus on the main messages.

**Reply:** We have made an effort to streamline sections of the discussion.

**Comment:** Adding scatterplots (e.g., VO2max vs. age, strength vs. age) would help visualize inter-individual variability.

**Reply:** Thank you for this notice. All figures have been revised accordingly, now including scatterplots.

**Comment:** Clarify technical terms (e.g., “O_2_ pulse,” “allometric scaling”) for non-specialist readers

**Reply:** In the revised manuscript, this is addressed in the Introduction- (Lines 50-52) and Allometric scaling paragraph of the Methods (Lines 200-201).

**Comment:** A light proofreading for grammar and flow would further improve readability.

**Reply:** Proofreading has been performed.

**4. Are the conclusions supported by the data?**

**Comment:** …the conclusions should remain descriptive rather than causal, given the cross-sectional design and absence of adjustment for potential confounders.

**Reply:** Revised as suggested. The conclusion is made more descriptive in the revised manuscript.

**Reviewer #3:**

Thank you for reviewing our manuscript. Your comments and concerns are hopefully addressed adequately. Please find the changes highlighted in the revised version of the manuscript.

**Comment:** How was it determined in the selection of participants who is able to perform exhaustive endurance and strength training and total testing. I recommend describing the selection criteria in more detail.

**Reply:** This is now elaborated on under the heading: Settings and participants in the Methods (Lines 88-93). In brief, everyone who fulfilled the inclusion criteria and were able to complete the physical tests were regarded as eligible.

**Comment:** I believe that the study would benefit greatly from reporting the differences in measured VO2max values in the general population. This would facilitate readers interpretation of the results given.

**Reply:** We do agree that key descriptives from the HUNT 3 study will make comparisons more accessible. This has been rectified in the Methods section, where a new paragraph and table (Table 2) display test specifics and characteristics of HUNT 3, respectively (Lines 147-154).

**Comment:** I recommend to deepen the discussion on the possible biochemical and psychosocial causes of the observed deficits.

**Reply:** These are no doubt important and interesting factors to discuss. Unfortunately, we have not collected any biochemical or psychosocial data, but we have addressed the possible causes of the observed deficits in the third paragraph of the discussion (Lines 375-381). This is certainly a relevant issue and should be implemented in future research.
