# Supplementary material for: Aerobic capacity and muscular strength in 179 Norwegian men and women aged 20–59 years with a substance use disorder: A cross-sectional study
Source: PLoS One. 2026 May 13;21(5):e0348707. doi: 10.1371/journal.pone.0348707 (PMC13170891; doi:10.1371/journal.pone.0348707)
Supplement: Attachment — Submitted filename: Response_to_Reviewers_auresp_2.docx [file pone.0348707.s005.docx]

**Dear Editor,**

Thank you for giving us the opportunity to re-submit a minor revision of our manuscript. Comments and concerns from the reviewers have been addressed with corresponding amendments in the manuscript. We do hope that the revised version of the manuscript is satisfactory for publication in PLOS ONE.

Sincerely,

Henrik Loe

**Response to Journal and Reviewers:**

**Reviewer #2:**

**Comment:** Data availability: The commitment to share raw data "freely if accepted" aligns with PLOS ONE policy in principle, but lacks specifics on repository (e.g., Figshare, Zenodo), DOI, or timeline—please clarify this before acceptance to fully comply

**Reply:** All relevant data will be included in **Supporting Information** files if manuscript is accepted for publication, an option in compliance with PLOS ONE data availability policy.

**Minor edits:**

**Comments:** Table 4 (and likely Table 5 for women) needs reformatting for clarity—ensure columns align properly, full data visible on one page if possible, and consistent CI notation (e.g., 45.9 [44.5–47.3])

**Reply:** These concerns have now been rectified in Table 4 (lines 270-273) and Table 5 (lines 293-296).

**Comment:** Some phrasing remains awkward (e.g., "Patients that fulfilled the inclusion criteria, and agreed to participate, was tested" should be "were tested"; "oxygen pumpedheartbeat" needs spacing/formatting).

**Reply:** The verb (was) is corrected to plural (were) (line 89). “oxygen pumped/heartbeat” changed to “oxygen pumped per heartbeat” (line 52)

**Comment:** Ensure consistent formatting for CIs and effect sizes across text/tables (e.g., η² values like "20.329 0,327" appear malformed).

**Reply:** Consistent formatting for CI implemented in Table 4 (lines 270-273) and Table 5 (lines 293-296) and effect sizes (lines 268-269 and lines 291-292).

**Comment:** The CPET ventilation note (two participants >180 L/min) is clear but could be more concise.

**Reply:** Amended the sentence with a more concise formulation as suggested (lines 138-139).

**Journal:**

No references have been added or retracted.
